# Cost-effectiveness analysis of cognitive behaviour therapy for treatment of minor or mild-major depression in elderly patients with type 2 diabetes: study protocol for the economic evaluation alongside the MIND-DIA randomized controlled trial (MIND-DIA CEA)

**DOI:** 10.1186/1471-2318-9-25

**Published:** 2009-07-01

**Authors:** Nadja Chernyak, Frank Petrak, Kristin Plack, Martin Hautzinger, Matthias J Müller, Guido Giani, Andrea Icks

**Affiliations:** 1 Institute of Biometrics and Epidemiology, German Diabetes Research Centre, Düsseldorf, Germany; 2 Department of Psychosomatic Medicine and Psychotherapy, LWL-Clinic Dortmund/Ruhr-University of Bochum, Dortmund, Germany; 3 Department of Clinical Psychology and Psychotherapy, Johannes Gutenberg University, Mainz, Germany; 4 Department of Psychology, Clinical Psychology and Psychotherapy, Eberhard Karls University, Tübingen, Germany; 5 Clinic for Psychiatry and Psychotherapy, Giessen, and Clinic for Psychiatry and Psychotherapy, Marburg-Süd, Germany

## Abstract

**Background:**

Depression and elevated depression symptoms are more prevalent in patients with type 2 diabetes than in those without diabetes and are associated with adverse health outcomes and increased total healthcare utilization. This suggests that more effective depression treatment might not only improve health outcome, but also reduce costs. However, there is a lack of evidence on (cost-) effectiveness of treatment options for minor and mild-major depression in patients with type 2 diabetes. In this paper we describe the design and methods of the economic evaluation, which will be conducted alongside the MIND-DIA trial (Cognitive behaviour therapy in elderly type 2 diabetes patients with minor or mild-major depression). The objective of the economic evaluation (MIND-DIA CEA) is to examine incremental cost-effectiveness of a diabetes specific cognitive behaviour group therapy (CBT) as compared to intensified treatment as usual (TAU) and to a guided self-help group intervention (SH).

**Methods/Design:**

Patients will be followed for 15 months. During this period data on health sector costs, patient costs and societal productivity/time costs will be collected in addition to clinical data. Person-years free of moderate/severe major depression, quality adjusted life years (QALYs), and cumulative costs will be estimated for each arm of the trial (CBT, TAU and SH). To determine cost-effectiveness of the CBT, differences in costs and effects between the CBT group and TAU/SH group will be calculated.

**Discussion:**

CBT is a potentially effective treatment option to improve quality of life and to avoid the onset of a moderate/severe major depression in elderly patients with type 2 diabetes and minor or mild-major depression. This hypothesis will be evaluated in the MIND-DIA trial. Based on these results the associated economic evaluation will provide additional evidence on the cost-effectiveness of CBT in this target population. Methodological strengths and weaknesses of the planned economic evaluation are discussed.

**Trial registration:**

The MIND-DIA study has been registered at the Current Controlled Trials Register (ISRCTN58007098).

## Background

Depression is a highly prevalent disorder with a substantial impact on quality of life and societal cost [1,2]. This applies in particular to patients with diabetes, since depression has been shown to be more prevalent among these patients as compared to those without diabetes [3-5]. Major depression and elevated depression symptoms were present, respectively, in 11 and 31% of individuals with diabetes according to a meta-analysis [4]. Previous research demonstrates that comorbid depression in patients with diabetes is associated with poor self care, i.e. adherence to medication, diet, exercise and smoking cessation [6-8], additive functional impairment and work disability [9], poorer glycaemic control [10], higher risk of microvascular and macrovascular complications [11,12], decreased quality of life [13], higher mortality [14,15], increased healthcare utilization and higher costs for other health conditions [7,16-19] as compared to patients with diabetes only.

Hence, the more effective depression treatment might not only improve health outcomes, but also reduce total health service utilization and therefore costs. Put differently, additional costs for improved depression treatment could be offset by reduction in other healthcare costs. For example, among older adults with diabetes in the IMPACT trial [20] systematic depression treatment had significant clinical benefit with no increase in overall healthcare costs.

Cost-effectiveness of antidepressive therapies has been mainly evaluated for major depression co-occurring with diabetes. However, since both major depression and depressive symptoms are associated with adverse outcomes in diabetes, there is a need to examine cost-effectiveness of treatment options for minor and mild-major depression as well, which is underscored by high prevalence of elevated depression symptoms in patients with diabetes and growing prevalence of diabetes due to the demographic change.

In what follows, we outline the economic evaluation of a cognitive behaviour therapy for treatment of minor or mild-major depression in elderly patients with type 2 diabetes that will be conducted alongside the MIND-DIA trial. Full details on the design and methods of the MIND-DIA trial will be provided elsewhere (Petrak et al, in preparation). Here, after giving a brief overview of the MIND-DIA trial, we focus on describing design and methods of the associated economic evaluation (MIND-DIA CEA).

## Methods/design

### Overview of the MIND-DIA trial

MIND-DIA trial is a multicentre, open, observer-blinded, randomized controlled trial, which will evaluate the effectiveness of a diabetes-specific cognitive behaviour group therapy (CBT) for treatment of a minor or mild-major depression in subjects 65–85 years of age with type 2 diabetes comparing it to intensified treatment as usual (TAU) or a guided self-help intervention "Successful aging with Diabetes" (SH). Approval for conducting this study was granted by the local Medical Ethics Committee (Ethikkommission bei der Landesärztekammer Hessen).

#### Study sample

A total number of 315 subjects will be included in the study. Patients will be recruited in approximately 20 centres specialising on diabetes treatment in Germany. In the participating centres all 65 to 85 year old patients with type 2 diabetes, who give informed consent, will be screened in a two-stage procedure (Patient Health Questionnaire (PHQ-9) [21] followed by the Structured Clinical Interview for the DSM IV (SCID) [22]). All patients with minor depression (adapted from the DSM-IV-TR research criteria: 3–4 symptoms rather than 2–4 symptoms are required, and a past history of major depression is not an exclusion criterion), or mild-major depression (5 to 6 depressive symptoms according to DSM-IV-TR criteria) will be included in the trial, provided that they meet other inclusion and exclusion criteria (inclusion of patients with diabetes mellitus type 2 diagnosed at least 6 months before entering the trial, residence near the institution where intervention and control treatments will take place (< 1 hour access); exclusion: e.g. history of schizophrenia, psychotic symptoms, or dementia, current antidepressant or relevant psychoactive medication).

#### Interventions

Patients included in the MIND-DIA trial will be randomized to one of the following interventions: CBT, SH or TAU. CBT is a manual-based diabetes-specific cognitive behaviour therapy, delivered by trained psychologists in small groups in an outpatient setting. The guided self-help group intervention 'Successful aging with Diabetes' (SH) with a focus on living and ageing with diabetes will be delivered by trained moderators (elderly care nurses, nurses or others). SH will include information regarding diabetes and ageing shared by members of the group. This intervention was conceptualized as a control condition for unspecific group effects (e.g. cohesion). Hence, no formal therapeutic aspects will be involved. In patients assigned to intensified usual care (TAU), treating physicians will be notified about the patient's minor or mild depression symptoms and cognitive function. Further, information on available therapeutic options will be provided to physicians. However, any treatment option may be chosen (antidepressant medication, psychotherapeutic interventions or no specific intervention), since care as usual for a minor depression is currently not formalized.

For patients of the CBT and SH groups the trial will comprise two phases: (1) 12 weeks of open-label therapy (weekly sessions of two hours each) and (2) one year maintenance phase, during which both group interventions (CBT and SH) will be reduced to one session per month. Usual diabetes therapy is not a part of the protocol and will be delivered by the treating physicians 'as usual'. For the first funding phase, the duration of the MIND-DIA trial is expected to be approximately 36 months. Recruitment of patients started in May 2009.

#### Clinical outcomes

Primary clinical endpoint will be the improvement in health-related quality of life (HRQoL) at one year follow-up after the 12 weeks of therapy as measured by the Mental Component Summary Score of the SF-36. Secondary clinical endpoints are Physical Component Summary score of the SF-36, improvement in HRQoL measured by the EQ-5D; reduction of minor or mild-major depression symptoms (QIDS-C-16); prevention of moderate/severe major depression (Depression module, Structured Clinical Interview for DSM-IV, SCID); improvement of glycaemic control (centrally measured HbA1c) and mortality.

#### Sample size calculation

The power calculation was based on expected differences in SF-36 z-scores. Differences of δ = 0.6 between CBT and TAU and of δ = 0.4 between CBT and SH were assumed. For the latter comparison 132 patients per intervention group were needed to detect a significant difference with a power of 90% (2-sided t-test, α = 0.05). Given 132 patients in the CBT group it is sufficient to enroll 51 patients in the TAU group to achieve a power of 95% for the comparison CBT vs. TAU. It is therefore planned to recruit a total number of 315 patients (132 in CBT, 132 in SH and 51 in TAU).

### Economic evaluation alongside the MIND-DIA trial (MIND-DIA CEA)

#### Outline of the economic analysis

The economic evaluation will be performed alongside the MIND-DIA clinical trial over the complete trial period. Costs and effects will be discounted at the rate recommended by the German guidance for economic evaluation issued by the Institute for Quality and Efficiency in the Health Care Sector (IQWiG), which is currently 3%. The economic evaluation will be undertaken from the perspective of the German statutory health insurance and from the societal perspective on costs. Accordingly, health sector costs, patient costs and societal productivity/time costs will be included in the analysis (Table 1).

**Table 1:** Endpoints, measurement instruments and time of data collection

**Endpoint**	**Questionnaire**	**Time of measurement (Month)**					
		**Baseline**	**M 3**	**M 6**	**M 9**	**M 12**	**M 15**

Person-years free of moderate or severe major depression	Depression module of the SCID	**x**	**x**				**x**

QALYs	EQ-5D SF-36	**x**	**x**	**x**	**x**	**x**	**x**

Health sector costs	Cost questionnaire	**x**		**x**		**x**	**x**

Patient costs	Cost questionnaire	**x**		**x**		**x**	**x**

Societal productivity/time costs	Cost questionnaire	**x**		**x**		**x**	**x**

The three outcomes estimated for the economic analysis will be person-years free of moderate/severe major depression, quality adjusted life years (QALYs), and cumulative cost accrued in each arm of the trial (CBT, TAU and SH). To estimate cost-effectiveness of CBT, an incremental cost-effectiveness ratio (ICER) will be calculated, i.e. the ratio of the difference in costs between CBT and TAU/SH divided by the difference in effects. Under statutory health insurance perspective on cost, ICER will be calculated using health sector costs only. Adopting the societal perspective, also patient costs and societal productivity/time costs will be added to the calculation of the ICER.

#### Estimating effects

##### Calculation of depression-free years

Person-years free of moderate/severe major depression will be calculated based on incidence of moderate or severe major depression in different treatment groups.

##### Translation of HRQoL measures into QALYs

For the purposes of economic analysis, measures comparable across disease areas are preferred. Most popular outcome measure for this purpose are the quality adjusted life-years (QALYs), which explicitly combine length and quality of life in a single measure, weighting survival (a set of health states) by utility scores. Utility weights reflect preferences for a particular health state and are measured on a scale from 0 to 1, where 0 and 1 represent death and full health, respectively [23,24]. Although the MIND-DIA study does not include a utility measurement as part of its protocol, it does include SF-36 und EQ-5D questionnaires measuring health related quality of life. Standardized algorithms exist to translate EQ-5D and SF-36 scores into utility weights suitable for calculation of QALYs [25,26].

The descriptive system of the EQ-5D allows for 243 unique health states. A preference-based scoring function can be used to convert the descriptive information to a summary index score (utility weight). More than 15 value sets are available for scoring the EQ-5D, based on rating scale and time trade-off (TTO) valuation derived from general population surveys in various countries (including the United Kingdom, Germany, and the United States) [27]. In this study the scoring function derived from a survey of the general population in Germany will be used to calculate utility weights from EQ-5D responses [28].

Brazier and colleagues reported work on deriving a reduced health status index from the SF-36 that they termed the SF-6D [29] and more recently, they have published an algorithm that allows the estimation of utility weights for all states of the SF-6D index [30]. Following this published algorithm, SF-36 scores observed in the trial will be converted to utility weights. Since the values underlying this algorithm were obtained in the United Kingdom, utilities derived from SF-36 scores will be only used to perform a sensitivity analysis.

In the MIND-DIA study, the SF-36 and EQ-5D instruments will be administered at baseline, after the intervention (at 3 months), and then quarterly during a 1 year follow-up (see Table 1), generating a maximum of six possible observations for each patient enrolled in the trial. QALYs will be calculated assuming linear interpolation between measurement points and calculating the area under the curve to give a QALY score per patient over the trial period [24].

#### Estimating costs: measurement and valuation of resource use

##### Measurement of resource use

Resource use directly associated with CBT and SH (e.g. patients' and staff time due to screening and treatment) will be derived from the therapy protocols. Information on the other healthcare consumption will be obtained from trial participants by means of a cost questionnaire, which was developed for this purpose and incorporated into the case report files of the MIND-DIA trial. The questionnaire will be administered before the intervention (baseline), at 6, 12, and 15 months of the trial and refers to the previous 6 or 3 (for the last assessment) months (see Table 1). The cost form includes structured no/yes questions on the utilization of different medical services under the following categories: primary care visits, visits to emergency departments, visits to specialists, hospital stays, medication, and other therapies/paramedical care. If patients indicate that they received specific medical care over the past 6 or 3 months, they are asked to specify the volume: e.g. number of contacts with healthcare providers, number and length of hospitalizations, types and dosage of obtained medications. In the cost questionnaire patients are also asked to indicate whether health care services obtained by them were paid by the health insurance or self-paid, which makes an assessment of out-of-pocket expenses possible.

Furthermore, (leisure) time losses will be measured by (i) registering the number of 'disability' days (days of reduced activity at home) with the cost questionnaire and (ii) estimating average times of receiving medical care from data on health services utilization in the trial groups. Note that the majority of patients enrolled into the trial are expected to be retirees. Hence, only (leisure) time losses and not productivity losses (i.e. time missed from work) are captured in the analysis.

##### Health sector costs

To estimate costs from the statutory health insurance point of view, direct healthcare resource use of the interventions and other reported healthcare utilization (consultations, hospital days, etc.) will be multiplied by unit costs/prices. Currently, there are no German guidelines for costing in economic evaluations containing standard unit costs. Hence, healthcare resource use will be valued by unit costs/prices obtained from published sources and official statistics for Germany (e.g. charges and rates from administrative databases, pharmacy retail prices).

##### Patient costs

To estimate patient costs, reported consumption of healthcare services paid out of pocket will be multiplied by unit costs/prices available from official statistics and from providers. Patient travel costs will be calculated based on the amount of health care utilization and on average distances to health care providers.

##### Societal productivity/time costs

Days of reduced activity at home and time of receiving medical care will be valued using average net wage rates, which represent opportunity costs of lost unpaid work and leisure according to the human capital approach.

#### Statistical analysis of costs and effects

Mean total costs, health sector costs, patient costs and productivity/time costs of interventions as well as corresponding cost differences between the CBT group and TAU/SH group will be calculated. Sampling uncertainty will be estimated using bootstrap procedure because cost data are non-normally distributed.

Incidence rates of moderate/severe major depression in all groups will be estimated, along with 95% confidence intervals. The incidence rate ratio (the incidence rate of moderate or severe major depression in the CBT group over the incidence rate in the TAU/SH group) will be analysed by regressing depression status at follow-up on the type of intervention in a Poisson model.

Effect in terms of QALYs will be analysed using linear regression on type of intervention and – if necessary – on baseline utility score, which has been shown to be important for the unbiased assessment of mean QALY differences between treatment groups [31].

#### Imputation of missing information on costs and effects

Data will be analysed according to the intention to treat principle. A multiple imputation approach based on propensity scoring will be used to account for missing information with regard to effects and costs. Baseline variables (e.g. age, gender, cost at baseline, etc.) will be entered into a logistic regression to predict the chance of a missing value [32,33]. Available data will be arranged into quintiles based on this predicted probability (propensity score) and a replacement value for missing data will be selected at random from the available data points within the same quintile. By choosing a value at random within the same quintile the principle of multiple imputations could be employed, whereby each missing value is replaced by m > 1 simulated values [34-36]. Each of *m *resulting data sets will be analysed as described above and combined to produce a single result that takes uncertainty in the imputation process into account.

#### Determining cost-effectiveness

If a significant impact of CBT on both effects and costs is demonstrated, ICER will be estimated in terms of costs per year free of moderate/severe major depression and per QALY gained. ICER will be estimated for the total cost (health sector costs plus patient costs plus societal productivity costs) and for the health sector costs (statutory health insurance perspective) separately. The non-parametric bootstrap method will be employed to generate confidence intervals around the ICER estimates derived from the study sample [37,38]. Uncertainty surrounding the ICER will also be presented on the cost-effectiveness plane [39,40] and as the cost-effectiveness acceptability curve [41,42].

#### Sensitivity analyses

Besides statistical uncertainty (sampling variation) with regard to costs and effects, every economic evaluation may contain some degree of data imprecision (e.g. resource costs/prices) and methodological controversy (e.g. derivation of utility weights, discount rate), which should be accounted for. To handle this type of uncertainty, sensitivity analysis is usually employed [23,24,43]. In the sensitivity analysis (uncertain) parameter(s) of the base-case analysis are varied to determine if changes in these parameters influence the results. Univariate sensitivity analyses will be performed by varying health service unit costs and utility weights (see Table 2). Further, in order to assess how a simultaneous change of several variables affects the cost-effectiveness ratio, a multivariate sensitivity analysis will be performed. To fully appreciate the potential influence of missing responses and of the imputation method chosen, sensitivity analyses examining the effect of alternative imputation methods (linear extrapolation and complete case analysis) will be conducted. When conducting sensitivity analysis, we will report both the revised point estimates and revised confidence intervals for costs, effectiveness, and cost-effectiveness that result from the sensitivity analyses.

**Table 2:** Summary of planed sensitivity analyses

**Parameter/methodological assumption**	**Base-case**	**Sensitivity analysis**
Utility weights for QALYs	derived from EQ-5D	derived from SF-36

Unit costs/prices of resource use	data from published sources and official statistics for Germany	varied within a plausible range

Imputation method	multiple imputation	linear extrapolation, complete case analysis

## Discussion

### Methodological considerations

#### Identification of resource use/attribution

Research has shown consistent association between depression symptoms and increased total healthcare utilization and costs in patients with diabetes, even after controlling for co-morbidities. Consequently, it is difficult to identify (and to measure) resource use related to depression. In the context of the trial, it might be argued that, if true randomisation is achieved, any differences in cost between treatment arms can be attributed to the study intervention [44]. Hence, data on utilization of a broad range of health services will be collected in the MIND-DIA trial. On the one hand, this approach allows avoiding the neglect of any unexpected changes in resource use related to the interventions being compared. On the other hand, however, it may complicate the detection of statistically significant difference in health service costs, since the latter have been shown to be highly variable and therefore to require larger overall sample sizes [44,45].

#### Measurement of resource use

Information on healthcare utilization other than CBT and SH sessions will be collected by self-report by means of the cost questionnaire. To our knowledge no standard and validated instruments for collecting resource use data in clinical trials are available in Germany. Hence, we developed a data collection instrument specifically for the MIND-DIA trial. The questionnaire was pilot tested, but has not yet been validated against other data sources. Recall bias may potentially occur, since resource use will be measured over the previous 6 or 3 months. However, there is no conclusive evidence regarding whether a prospective (a cost diary) or a retrospective (a questionnaire) instrument should be better applied and regarding an appropriate recall interval (44). Van den Brink et al. found that for the assessment of healthcare utilization in economic evaluations alongside clinical trials, a cost questionnaire may replace a cost diary for recall periods up to 6 months [46] and that such patients' self-reports are a valid source of data on days of hospitalization and out-patient visits, whereas costs of medication may be underestimated [47].

#### Time costs

In this study days of reduced activity at home and time of receiving medical care will be monetary valued using average net wage rates representing opportunity cost of unpaid work and leisure according to the human capital approach. It is contentious, however, whether lost (leisure) time should be measured in terms of costs or effects [23,24,48,49]. In particular, lost (ability to enjoy) leisure time might as well be reflected in QALYs, since health related quality of life instruments, e.g. the EQ-5D and the SF-36, explicitly ask about problems in performing leisure activities and social activities [48]. Thus, double counting may occur. We nevertheless decided to capture time costs in the numerator of the cost-effectiveness ratio, when adopting the societal perspective, because it is unclear to what extent changes in (leisure) time are measured by QALYs and the true societal loss may be underestimated otherwise. Furthermore, this approach will help to avoid some unpalatable equity implications, since time missed from work due to treatment or illness is most often captured in monetary terms.

## Conclusion

Depression and depression symptoms co-occurring with type 2 diabetes are highly prevalent and associated with a wide range of adverse outcomes, including less effective self-care, more severe physical symptoms, greater functional impairment and disability as well as increased healthcare utilization and expenditure. However, there is a lack of evidence on effectiveness of treatment options for minor or mild-major depression co-occurring with diabetes. Therefore, the MIND-DIA trial will evaluate the effectiveness of cognitive behaviour therapy for treatment of sub-threshold depression in elderly patients with type 2 diabetes, comparing it to a self-help group intervention and to usual care.

The negative impact of co-morbid depression on both health effects and total costs suggests that the more effective treatment of depression might not only improve health outcomes in patients with diabetes, but also change the pattern of health services use and therefore total healthcare costs. Costs for depression treatment might be balanced by reduction in other healthcare utilization or even lead to savings in total costs. Thus, besides testing the effectiveness of CBT as a treatment option to improve quality of life and to avoid the onset of a moderate or severe major depression in elderly patients with type 2 diabetes and minor or mild-major depression, cost-effectiveness of the CBT should be examined as well. The economic evaluation conducted alongside the MIND-DIA trial will provide additional evidence on whether CBT is a cost-effective strategy in this target group. Importantly, since patients are followed for 15 months, long-term incremental costs and effects of the CBT will be captured.

## Competing interests

The authors declare that they have no competing interests.

## Authors' contributions

AI and NC developed the design and methods for the economic evaluation alongside the clinical trial. NC wrote the manuscript. GG gave support relating to the statistical analysis. FP is the coordinator and principal investigator of the clinical trial. KP, MH, and MM are the coordinating investigators of the clinical trial. All co-authors read, edited, and approved the final manuscript. All authors participated in the work sufficiently to take public responsibility for respective parts of the paper.

## Pre-publication history

The pre-publication history for this paper can be accessed here:


